# Development of a practice-based score to predict extended duration of proton beam therapy session in pediatric patients

**DOI:** 10.1007/s12094-025-03972-4

**Published:** 2025-06-19

**Authors:** José M. Fernández-de Miguel, Miguel Ángel García-Aroca, Ignacio Manrique Yera, Felipe A. Calvo, María Aymerich de Francesci, Jose Manuel Álvarez Avello, Elena Panizo Morgado, Jorge M. Núñez-Córdoba

**Affiliations:** 1 Department of Anaesthesia and Critical Care, Clínica Universidad de Navarra, Madrid, Spain; 2 Department of Anesthesia and Intensive Care, HCD “Gómez-Ulla”, Madrid, Spain; 3 Operations Department and Protontherapy Unit, Clínica Universidad de Navarra, Madrid, Spain; 4 Department of Radiation Oncology, Cancer Center, Clinica Universidad de Navarra, Madrid, Spain; 5 Department of Anaesthesia and Critical Care, Clínica Universidad de Navarra, Madrid, Spain; 6 Department of Pediatric Oncology, Clínica Universidad de Navarra, Madrid, Spain; 7 Research Support Service, Central Clinical Trials Unit, Clínica Universidad de Navarra, Pio XII, 36, 31008 Pamplona, Spain; 8 Institute of Data Science and Artificial Intelligence, University of Navarra, 31009 Pamplona, Spain; 9 Department of Health Sciences, Public University of Navarra, 31008 Pamplona, Spain

**Keywords:** Pediatric cancer, Proton beam therapy, Treatment planning

## Abstract

**Purpose:**

Due to the labor intensity demanded by proton beam therapy (PBT) in pediatric patients, information on operational procedures related to efficiency is crucial to optimize quality and safety. We aimed to identify patient factors that affect the duration of the pediatric PBT session and to develop an easy-to-use predictive score of extended duration.

**Methods/patients:**

This is an observational retrospective cohort study in an academic medical centre, between May 2020 and February 2024. Seventy seven ASA III pediatric patients treated with PBT were recruited.

**Results:**

The mean age was 4.8 years [standard deviation (SD): 2.1] and 52% were women. The mean duration of the PBT session was 50 min (SD: 17). Extended duration of the PBT session (> 45 min) occurred in 39 patients (51%). Five predictors of extended duration were selected for the final prediction model. In the multivariable model, an age > 45 months showed a near eightfold increased odds of extended duration [Odds ratio (OR): 7.76, 95% confidence interval (95% CI) 1.63–36.99, *P* = 0.010]. The OR (95% CI) for long-term venous access, no recurrent tumors, hydrocephalus, and craniospinal location were 5.91 (1.47 to 23.79), 3.81 (0.67 to 21.69), 3.79 (0.90 to 15.97), and 2.59 (0.69 to 9.76), respectively. This five-variable model was used to build a nomogram-based score with an area under the receiver operating characteristic curve of 0.84 (95% CI 0.76–0.93).

**Conclusions:**

A simple nomogram based on readily available pretreatment data has potential for planning pediatric PBT standard clinical expert practice.

## Introduction

According to the American Cancer Society, cancer is the second leading cause of death among children aged 1–14 years after accidents in the United States [1]. The projections for 2025 reveal that more than 9500 children (aged birth to 14 years) will be diagnosed with cancer, and more than 1000 children will die from the disease. Despite these worrying estimates, there is also evidences of progress. The overall invasive cancer incidence rate in children declined slightly from 2015 through 2021 by 0.8% per year and cancer mortality in these patients declined steadily from 6.3 per 100,000 in 1970 to 1.9 per 100,000 in 2020–2022 [1]. Moreover, the 5‐year relative survival rate for all cancers combined improved from 58% for diagnoses during the mid‐1970s to 85% during 2014 through 2020 in children [1]. In the context of these survival improvements, the focus on quality of life in pediatric cancer population gains importance. A key element to protect quality of life of pediatric cancer survivors is to reduce long-term sequelae induced by treatment.

Proton beam therapy (PBT) has emerged as an efficient technology for pediatric patients that require radiotherapy due to the potential of PBT to mitigate treatment-associated toxicities [2–5]. However, PBT for pediatric patients has been reported to be two to four more labor-intensive than for adult patients [6]. Continued efforts to improve and optimize pediatric PBT are needed. The variability in time duration of pediatric PBT sessions may be substantial, suggesting that specific factors may be a target for improved care [6]. The identification of factors influencing the time duration of a pediatric PBT session is relevant for efficient and productive planning purposes. No study has evaluated the multifactorial integrated scenario that specifically involves the duration of the pediatric PBT session.

The objectives of this study were to identify patient factors that affect the duration of the pediatric PBT session and to develop a simple and easy-to-use predictive nomogram-based score useful for planning pediatric PBT standard clinical expert practice. This may help optimize quality and safety of pediatric PBT and, consequently, improve satisfaction of patients, family, staff, and physicians.

## Materials and methods

This is an observational retrospective cohort study of ASA III pediatric patients that were treated with PBT at Clínica Universidad de Navarra (Madrid, Spain) between May 2020 and February 2024. The Clinica Universidad de Navarra Proton Therapy Unit is the first commercial synchrotron equipment for PBT operating in Europe and the fourth commercial 360º gantry available for clinical use worldwide [7].

The full details of the anesthetic management has been described elsewhere [8]. Briefly, patients and their parents were received by a pediatric nurse when arrived to the PTB center, where all staff (anesthesiologists, anesthesia nurses, pediatric oncologist, radiation oncologists and radiation therapy technicians) were committed to create an environment as comfortable as possible for patients and family members. Thus, patients were allowed to play with toys and use devices to watch and/or listen their favourite cartoons and/or songs. Anesthesia was induced in the treatment room. The equipment of the treatment room was similar to operating rooms, including usual instrumentation, ventilatory support, and medications. The anesthesia procedure adhered to the Helsinky Declaration on Patient Safety and Quality in Anesthesiology [9]. All participating anesthesiologists followed the same standardized anesthetic protocol. Parents were allowed to participate in the inhalational anesthetic induction applying the face mask, with the patient on the table or sat on the parent's lap. This approach was followed as a way of minimizing anxiety in patients and improving tolerance. A mixture of 70% nitrous oxide (N2O) and 30% oxygen was administered via the face mask. At this stage, only exhaled gases and digital pulse oximeter were included in the anesthetic monitoring. When reaching 70% exhaled N2O concentration, the patient was laid down on the treatment table and an anesthesia nurse completed the basic ASA monitoring standards [10]. Then, the initial gaseous mixture was substituted by one based on oxygen and air at 50%, with sevoflurane at 8%. Afterwards, the sevoflurane concentration was reduced at the discretion of the anesthesiologist in charge. When the eyelash reflex was lost, a laryngeal mask airway (LMA, Ambu®Aura-i™) was inserted. Finally, the concentration of sevoflurane was reduced to a minimum alveolar concentration (MAC) adapted to the patient's age [11, 12], with oxygen at 30%, the radiation technician placed the thermoplastic mask over the target patient´s body part, and the patient was covered with a thermal blanket. At the end of the anesthetic induction, the patient was ready to receive the treatment. Patients maintained spontaneous ventilation during all anesthetic management. Once the patient was correctly positioned, the staff left the treatment room. This room is equipped with an anesthetic gas scavenging system and video cameras for anesthetic monitoring. After finished session, the thermoplastic mask and LMA were removed without modifying the gas concentration used during the procedure. Then, the patient was moved to lateral position, and transferred to the anesthesia recovery room with pulse oximeter monitoring. The standard PBT procedure includes to perform a cone–beam CT daily for each volume to be treated.

On the basis of clinical expert consensus, data from the following variables were collected to identify potential predictors of extended duration of PBT session: age, sex, weight (percentile), country, type of tumor, tumor location, recurrent tumor, hydrocephalus, tracheostomy, gastrostomy, neurological pathology, swallowing disorders, pretreatment surgery, pretreatment chemotherapy, pretreatment radiotherapy, chemotherapy during treatment, and long-term venous access.

The PBT duration session was defined as the time between patient entering and exit the treatment room. A time duration for a session of PBT greater than 45 min was considered as extended duration (median split method). This value was available in the PBT monitors for treatment prescription.

### Statistical analysis

All available patients from the study period that fulfill eligibility criteria were included for analyses. No formal sample size estimations were performed. No missing data imputation method was used for this analysis. Mean, standard deviation (SD), median and interquartile range (IQR) for continuous variables and frequencies for categorical variables were calculated. Age was categorized to facilitate clinical use of the predictive nomogram using the Youden Index procedure (≤ 45 and > 45 months). Group comparisons among patients with extended duration of the PBT session versus duration ≤ 45 min were carried out using Pearson's chi-squared test or Fisher exact test for categorical variables and *t* test for continuous variables. Those variables that showed statistically significant differences were selected as potential candidate predictors. The associations of potential candidate predictors with extended duration were evaluated using binary logistic regressions. A multivariable logistic regression model for extended duration was built with all candidate predictors as independent variables. Final variable selection was accomplished through stepwise backward logistic regression with *P* < 0.2 used as the criterion for retaining predictors in the model. A simplified scoring system was developed with the predictors in the final model. The discriminative ability of the nomogram was quantified by the area under the receiver operating characteristic curve (AUC). An optimal cutoff point for the simplified nomogram score was determined using the Youden Index. We then calculated the sensitivity (Se), specificity (Sp), positive predictive value (PPV), and negative predictive value (NPV) for this cutoff point, with corresponding 95% confidence interval (95% CI). The two-sided significance level was set at p < 0.05. All statistical analyses were performed using Stata (StataCorp. 2023. Stata Statistical Software: Release 18. College Station, TX: StataCorp LLC) and R software (version 4.5.0).

## Results

A total of 77 ASA III pediatric patients treated with PBT were included in the study. The mean age was 4.8 years (SD: 2.1) and 52% were women. The mean duration of the PBT session was 50 min (SD: 17, median: 45, IQR: 37–59). Extended duration of the PBT session (> 45 min) occurred in 39 patients (51%). Characteristics of the study participants across categories of duration of the PBT session appear in Table 1. The distribution of demographic and clinical characteristics were compared across the two groups of duration of the PBT session. Age, CNS tumor, spinal cord location, craniospinal location, recurrent tumor, hydrocephalus, and long-term venous access, showed statistically significant differences between groups. These seven variables were considered as acceptable candidate predictors for further exploration.

**Table 1:** Patient characteristics according to duration of the PBT session

	All patients (*n* = 77)	Duration of the PBT session		*P* value
		≤ 45 min (*n* = 38)	> 45 min (*n* = 39)	
Age (years), mean (SD)	4.8 (2.1)	4.0 (1.8)	5.6 (2.1)	0.001
Age > 45 months, *n* (%)	56 (72.7)	21 (55.3)	35 (89.7)	0.001
Women, *n* (%)	40 (51.9)	22 (57.9)	18 (46.2)	0.303
Weight percentile, mean (SD)	37.5 (31.2)	37.9 (29.8)	37.1 (33.0)	0.921
Country, *n* (%)				
Spain	52 (67.5)	26 (68.4)	26 (66.7)	0.869
Other	25 (32.5)	12 (31.6)	13 (33.3)	
Tumor, *n* (%)				
CNS tumor	49 (63.6)	19 (50.0)	30 (76.9)	0.014
Sarcoma	19 (24.7)	12 (31.6)	7 (17.9)	0.165
Neuroblastoma	5 (6.5)	3 (7.9)	2 (5.1)	0.675
Wilms'tumor	2 (2.6)	2 (5.3)	0 (0)	0.240
Others	2 (2.6)	2 (5.3)	0 (0)	0.240
Location, *n* (%)				
Brain	61 (79.2)	29 (76.3)	32 (82.1)	0.535
Thorax	3 (3.9)	2 (5.3)	1 (2.6)	0.615
Abdomen	11 (14.3)	5 (13.2)	6 (15.4)	0.780
Spinal cord	36 (46.8)	10 (26.3)	26 (66.7)	0.001
Craniospinal	33 (42.9)	8 (21.1)	25 (64.1)	< 0.001
Recurrent, *n* (%)	13 (16.9)	10 (26.3)	3 (7.7)	0.029
Hydrocephalus, *n* (%)	33 (42.9)	11 (28.9)	22 (56.4)	0.015
Tracheostomy, *n* (%)	1 (1.3)	0 (0)	1 (2.6)	1.000
Gastrostomy, *n* (%)	10 (13.0)	4 (10.5)	6 (15.4)	0.737
Neurological pathology, *n* (%)	44 (57.1)	18 (47.4)	26 (66.7)	0.087
Swallowing disorders, *n* (%)	15 (19.5)	5 (13.2)	10 (25.6)	0.167
Pretreatment surgery, *n* (%)	66 (85.7)	33 (86.8)	33 (84.6)	0.780
Pretreatment chemotherapy, *n* (%)	57 (74.0)	28 (73.7)	29 (74.4)	0.946
Pretreatment radiotherapy, *n* (%)	12 (15.6)	8 (21.1)	4 (10.3)	0.192
Chemotherapy during treatment, *n* (%)	18 (23.4)	7 (18.4)	11 (28.2)	0.310
Long-term venous access, *n* (%)	57 (74.0)	24 (63.2)	33 (84.6)	0.032
Central venous catheter	1 (1.3)	1 (2.6)	0 (0)	0.494
Hickmann	6 (7.8)	3 (7.9)	3 (7.7)	1.000
PICC	3 (3.9)	2 (5.3)	1 (2.6)	0.615
Port-a-Cath	50 (64.9)	20 (52.6)	30 (76.9)	0.026

*CNS* central nervous system, *PBT* proton beam therapy, *PICC* peripherally inserted central catheter, *SD* standard deviation

**Fig. 1:**
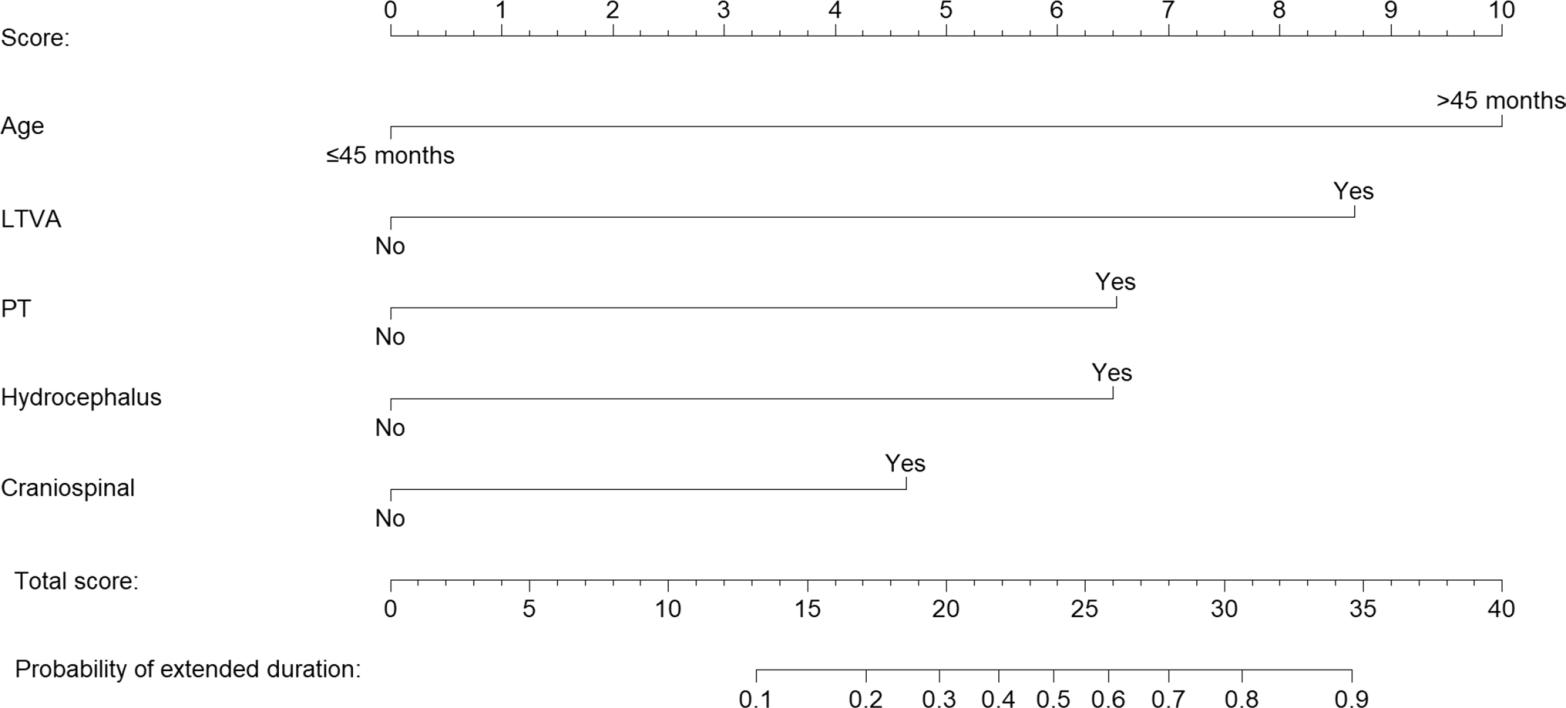
Nomogram using 5 readily available clinical characteristics to predict extended duration of proton beam therapy session in pediatric patients. LTVA: long-term venous access. PT: primary tumor (no recurrent tumor). To calculate probability of extended duration, first determine score for each factor by drawing vertical line from that factor to “score”. Then sum all individual values and draw vertical line from “total score” to probability of extended duration

**Fig. 2:**
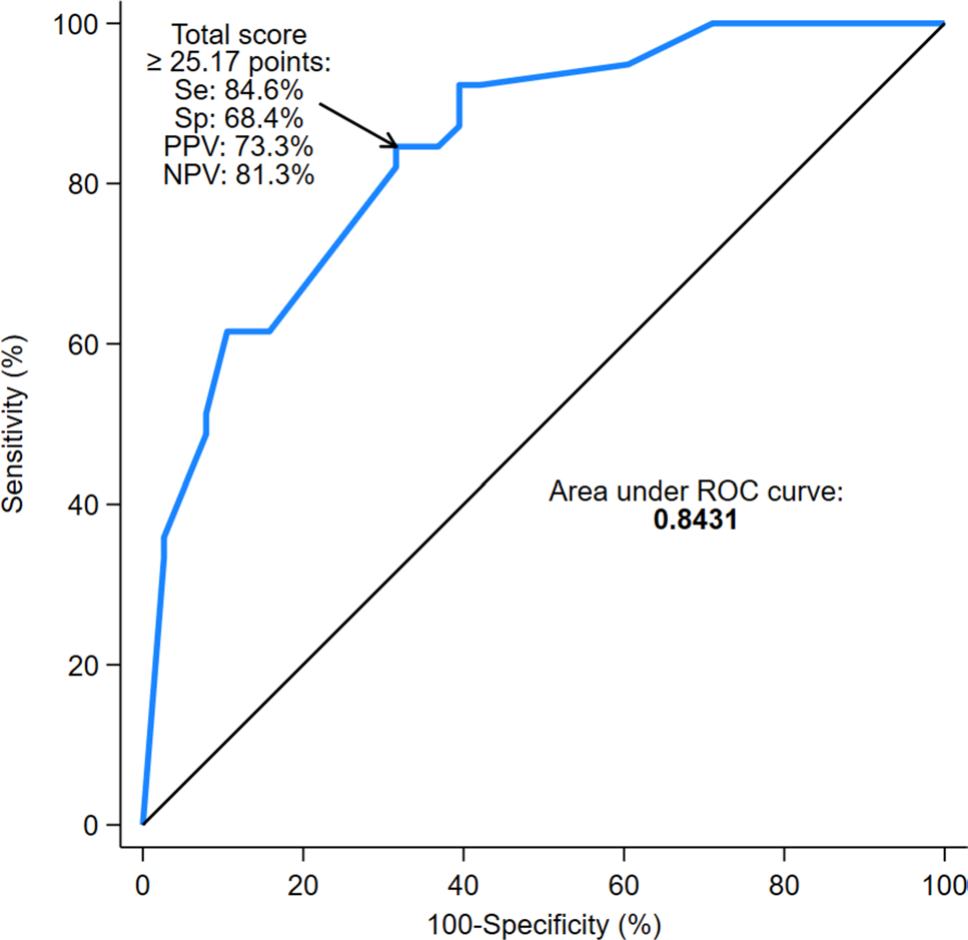
ROC curve for the five-variable nomogram to predict extended duration of proton beam therapy sessions. *NPV* negative predictive value, *PPV* positive predictive value, *Se* sensitivity, *Sp* specificity

Table 2 shows the unadjusted associations with extended duration of the seven candidate predictors. All these predictors showed a statistically significant association with extended duration in the univariable analyses. The estimated unadjusted-OR (95% CI) ranged from 3.18 (1.24–8.17) for hydrocephalus to 7.08 (2.10–23.90) for age > 45 months. After the stepwise procedure for variable selection, a final predictive model for extended duration was built with five selected predictors. The main effects of the selected predictors are presented as OR (95% CI) in Table 3. In the multivariable model, an age > 45 months showed a near eightfold increased odds of extended duration (OR: 7.76, 95% CI 1.63–36.99, *P* = 0.010). Furthermore, multivariable-adjusted OR (95% CI) for extended duration associated with long-term venous access, no recurrent tumors, hydrocephalus, and craniospinal location were 5.91 (1.47 to 23.79), 3.81 (0.67 to 21.69), 3.79 (0.90 to 15.97), and 2.59 (0.69 to 9.76), respectively. Then, this five-variable model was used to build a nomogram to ease its use in practice (Fig. 1). The AUC of the predictive nomogram was 0.84 (95% CI 0.76–0.93) (Fig. 2). At the cutoff of 25.17 total points based on the Youden Index, the five-variable nomogram yielded a Se of 84.6% (95% CI 69.5–94.1%), Sp of 68.4% (95% CI 51.3–82.5%), PPV of 73.3% (95% CI 58.1–85.4%), NPV of 81.3% (95% CI 63.6–92.8%). Patients may be categorized into two risk groups using this cutoff point of the prediction nomogram for extended duration of the PBT session (Fig. 2).

**Table 2:** Univariable analyses of candidate predictors for extended duration of PBT

Candidate predictor	Unadjusted OR (95% CI)	*P* value
Age > 45 months	7.08 (2.10–23.90)	0.002
CNS tumor	3.33 (1.25–8.88)	0.016
Spinal cord	5.60 (2.10–14.95)	0.001
Craniospinal	6.70 (2.42–18.53)	< 0.001
No recurrent tumors	4.29 (1.08–17.06)	0.039
Hydrocephalus	3.18 (1.24–8.17)	0.016
Long-term venous access	3.21 (1.08–9.56)	0.036

*95% CI* 95% confidence interval, *CNS* central nervous system, *OR* odds ratio

**Table 3:** Predictors of extended duration of PBT identified by multivariable logistic regression analysis

Predictor		Multivariable OR (95% CI)	*P* value
Age	≤ 45 months	Ref	–
	> 45 months	7.76 (1.63–36.99)	0.010
Long-term venous access	No	Ref	–
	Yes	5.91 (1.47–23.79)	0.012
No recurrent tumors	No	Ref	–
	Yes	3.81 (0.67–21.69)	0.131
Hydrocephalus	No	Ref	–
	Yes	3.79 (0.90–15.97)	0.070
Craniospinal	No	Ref	–
	Yes	2.59 (0.69–9.76)	0.161

*95% CI* 95% confidence interval, *OR* odds ratio, *Ref.* reference

## Discussion

There is urgent need to identify the best evidence on characteristics of PBT in pediatric cancer and treatment outcomes [14]. Due to the labor intensity demanded by this technique, information on operational procedures related to efficiency is also of paramount relevance. To our knowledge, this is a pioneer analysis to explore patient and disease factors with potential impact in the treatment time of pediatric undergoing PBT, in an effort to develop a practise-based nomogram to predict extended duration of PBT session in pediatric patients. This nomogram is an easy-to-use tool that uses readily available pretreatment data that can help providers to improve pediatric PBT planning and efficiency.

In this study, the estimated mean duration of the PBT session was apparently longer than previous reported research. We estimated an overall mean treatment time of 50 min (SD: 17; range: 5–109 min) for 77 pediatric patients under sedation, aged from 8 months to 10.2 years (MEAN age: 4.8 years, SD: 2.1 years). Mizumoto et al. [6] evaluated the treatment time of 32 pediatric patients, 12 of whom were treated while under sedation. The mean treatment time for these pediatric cases with sedation was 30.1 min (range: 10–145 min). These patients were seemingly younger (mean age: 3 years; range: 1–5 years) than those included in this study, which could explain differences in treatment times. Apart from age, no other demographic or clinical characteristics of these patients were reported, which preclude the possibility of comparing and hypothesizing other potential explicative factors for the difference in treatment time.

We identified five main factors that were independently associated with higher risk of extended duration of the PBT session. These factors include age, long-term venous access, hydrocephalus, and primary tumors (no recurrent). The age emerged as the most influential factor of treatment time, with older patients having longer treatment duration. This seems consistent with the aforementioned higher means of age and treatment time observed in this study compared with those reported by Mizumoto et al. [6].

A hypothetical explanation for the association between age and treatment time could be based on the differences in sedation requirements between younger and older pediatric patients. In the study carried out by Mizumoto et al. [6] was observed that the mean treatment times were 20.7 min for pediatric cases without sedation and 30.1 min for pediatric cases with sedation. However, the mean ages of the pediatric patients were 3 years (range: 1–5 years) for those treated with sedation and 5 years (range: 3–7) for those treated without sedation. In this study, the potential influence of sedation requirement on the association between age and treatment time was minimized, as all patients were with sedation.

The presence of long-term venous access was another factor related to extended duration of the treatment. In this study, 74% of patients had long-term venous access, being Port-a-Cath the most common option (87.7%), followed by Hickmann (10.2%), PICC (5.3%), and central venous catheter (1.8%). Hydrocephalus, no recurrent tumors, and craniospinal location also related to higher treatment time. Evidence to substantiate these associations are scarce or not available, although some hypotheses could be formulated, such as the compliance with a more demanding infection prevention protocol in long-term venous access cases, or the treatment of patients with larger tumor size in no recurrent cases. The influence of the preparation process on the treatment time has been previously reported [15]. A large impact of this factor may be rule out in this study, as the preparation process follow the same protocol for all patients.

Another factor that may be important, but not captured in our predictive nomogram, is the type of the tumor. In this evaluation, patients with CNS tumor were apparently more prone to extended duration of the PBT session. Likewise, spinal cord tumors seem to predispose to longer treatment time. Both factors are associated with the craniospinal location, which was included in the final predictive model.

This study demonstrated that a five-variable nomogram may have acceptable performance to discriminate PBT extended duration in pediatric patients. Thus, this easy-to-use system can help guide pediatric PBT management.

Some limitations of our study deserve mention. First, our evaluation was retrospective and the availability of information on other potential factors influencing the treatment time may be limited. However, we had access to accurate and detailed medical records for the main potential predictors targeted to be assessed in this study. Second, the evaluation focused on a population of pediatric patients under sedation. Therefore, caution is warranted when generalizing these results to pediatric patients treated without sedation. Third, our sample comprises a single expert institution with a specific technology and protocolized approach, which might not be generalizable to a broader population. Finally, the time for the PBT session was defined as the treatment time, excluding the transportation time (for example, from the pediatric ward to the PBT facility). This should be noted for an appropriate interpretation of these results.

In conclusion, this study estimated a mean pediatric PBT time of 50 min and derived a five-variable nomogram with potential for prediction of pediatric PBT extended duration. These results may be of great interest for planning pediatric PBT sessions. Although further studies refining the proposed nomogram in larger data sets and exploring its external validation are warranted before any recommendation regarding its routine clinical use, this study will hopefully contribute to improving pediatric PBT efficiency.

## Data Availability

The data that support the findings of this study are available from the corresponding author, upon reasonable request and institutional permission.
